# Sexual activity and sexual health among young adults with and without mild/moderate intellectual disability

**DOI:** 10.1186/s12889-018-5572-9

**Published:** 2018-05-29

**Authors:** Susannah Baines, Eric Emerson, Janet Robertson, Chris Hatton

**Affiliations:** 1 Centre for Disability Research, Lancaster University, Lancaster, LA1 4YT UK; 2 Centre for Disability Research and Policy, Faculty of Health Sciences, University of Sydney, Sydney, NSW 2006 Australia

**Keywords:** Sexual health, Sexual activity, Intellectual disability, Intellectual impairment, Borderline intellectual functioning, Cognitive ability

## Abstract

**Background:**

There is widespread concern about the sexual ‘vulnerability’ of young people with intellectual disabilities, but little evidence relating to sexual activity and sexual health.

**Method:**

This paper describes a secondary analysis of the nationally representative longitudinal Next Steps study (formerly the Longitudinal Survey of Young People in England), investigating sexual activity and sexual health amongst young people with mild/moderate intellectual disabilities. This analysis investigated family socio-economic position, young person socio-economic position, household composition, area deprivation, peer victimisation, friendships, sexual activity, unsafe sex, STIs, pregnancy outcomes and parenting.

**Results:**

Most young people with mild/moderate intellectual disabilities have had sexual intercourse by age 19/20, although young women were less likely to have sex prior to 16 than their peers and both men and women with intellectual disabilities were more likely to have unsafe sex 50% or more of the time than their peers. Women with intellectual disabilities were likely to have been pregnant and more likely to be a mother.

**Conclusion:**

Most young people with mild/moderate intellectual disabilities have sex and are more likely to have unsafe sex than their peers. Education and health services need to operate on the assumption that most young people with mild/moderate intellectual disabilities will have sex.

## Background

Intellectual disability refers to a significant general impairment in intellectual functioning that is acquired during childhood. It is commonly defined as scoring more than two standard deviations below the population mean on tests of general intelligence (IQ < 70). While estimates of the prevalence of intellectual disability vary widely [1], it has been estimated that approximately 2% of the adult population of England have an intellectual disability [2].

People with mild or moderate intellectual disabilities are often ineligible for assistance from local authority services but still experience considerable difficulties due to their disabilities. The follow up to the National Child Development Study Cohort suggested that adults with mild to moderate learning disabilities are more likely than their peers to live with their parents, be unemployed, have literacy and numeracy problems and to experience high levels of psychological distress [3].

Although there is an increasing awareness and recognition of the rights of people with intellectual disabilities to live ordinary lives and make their own decisions, sexuality remains an area where these freedoms are often limited compared to other disabled people or the general population [4]. This may in part be due to concerns from families and carers about vulnerability to exploitation, sexually transmitted diseases, and pregnancy [5], but may also reflect societal stigma and residual infantilising attitudes towards people with intellectual disabilities [6, 7].

People with intellectual disabilities are more likely to have limited social networks with fewer friends and socialise less than the general population [8, 9]. Requiring support with everyday tasks and living with parents or with carers, may also limit the opportunities for people with intellectual disabilities to either create independent friendships or develop a sexual relationship.

In a study that looked at how sexuality and sex education is viewed by mothers of adolescents with intellectual disabilities compared with mothers of adolescents from the general population, Pownall et al. [5] found mothers of adolescents with intellectual disabilities discussed fewer sexual topics, started these discussions at a later age and expressed more concerns about sexual vulnerability than mothers of other adolescents. Withholding information from their adolescent children was felt by families to be in their best interests to protect them from possible exploitation. They also reported that their children did not have the same sexual desires as other young people of their age and were uninterested in forming sexual relationships. This was particularly true for mothers of girls who they felt to be particularly vulnerable, presumably due to the risk of pregnancy [5].

For people with intellectual disabilities who rely on care staff to facilitate their everyday lives, staff attitudes may affect their freedom and opportunities to engage in friendships and relationships. Socially conservative views may impact on their willingness to acknowledge or support people with intellectual disabilities with their sexual health and relationships [7]. The authors also noted that staff who felt that people with intellectual disabilities should not engage in sexual relationships were unlikely to facilitate such a relationship. Research by Evans et al. has suggested that such differences were often generational and younger carers were more likely to acknowledge and facilitate intimate relationships [6]. However staff were found to be uncertain as to whether service users had rights to privacy, for example whether allowing two service users to be alone together was ‘allowed’.

Young people with intellectual disabilities have lower levels of sexual knowledge compared with their peers from the general population [10], even in localities with a strong emphasis on sex education programmes [4]. The latter author, McCabe, noted that people with intellectual disabilities were less likely to discuss sexual issues with family or friends leading to a lack of normalisation and consolidation of knowledge and concluded that, as well as limiting sexual knowledge, this may convey a negative message to people with intellectual disabilities about their sexuality.

Jahoda and Pownall had similar findings when they conducted a study looking at sexual understanding and social networks with 30 young people with mild intellectual disabilities and 30 peers from the general population [10]. They found that teenagers from the general population had a better understanding of sexual matters than those with intellectual disabilities, but that there was a gender difference. Young women from the general population had a better understanding than young men from the general population but young men with intellectual disabilities had a better understanding than young women with intellectual disabilities. The reason for this finding is uncertain. It may reflect the sheltering of young women from sexual knowledge expressed during Pownall’s research with mothers [5]; limiting any talk or discussion around sex is seen as a means of keeping their vulnerable daughters safe.

It may also reflect how having limited social networks can contribute to low levels of sexual knowledge and understanding [5, 10]. Lower levels of friendship and participation in social groups by people with intellectual disabilities are common [8, 9] and as learning about sex through discussions with peers is common amongst adolescents, young people with intellectual disabilities may miss out [10]. In Jahoda and Pownall’s research adolescents with intellectual disabilities were found to hold more misconceptions than young people from the general population including misunderstandings about sex such as believing that you cannot get pregnant the first time you have intercourse [10]. Incorrect information is unable to be corrected without further discussion between the young person and more knowledgeable friends or family [4].

Children and young people with intellectual disabilities, as well as having limited social networks, are more likely to be the victims of bullying or other forms of peer victimisation [11]. Kavanagh et al.’s research in Australia found bulling and social victimisation to be more prevalent among children with parents with low education, which is consistent with previous research that those in more challenging socio-economic positions may be more likely to be bullied [12].

It does not always follow that good knowledge about sex and contraception translates into safe sex. In a small qualitative study men with mild intellectual disabilities were found to engage in risky sexual practices despite having good sexual knowledge [13]. People with intellectual disabilities looking for correct information on sex and contraception may have difficulties finding a family planning clinic that caters for their needs. An audit of sexual health clinics in N. Ireland revealed that clinics were very poorly prepared to deal with people with developmental or intellectual disabilities, with few clinics providing specific services to people with intellectual disabilities [14].

There is less research that examines the sexual experiences of people with intellectual disabilities, as opposed to sexual knowledge. McCabe found that people with intellectual disabilities were less likely to have had sexual experiences than disabled people or young people from the general population. This also held true for all forms of sexual contact such as kissing or hugging naked but also for lower levels of intimacy such as hugging (fully clothed) or holding hands [4]. In an earlier study McCabe and Cummins found that, compared to college students, participants with mild intellectual disability were less likely to have experienced sexual intimacy or intercourse but, if they had engaged in sexual intercourse, were more likely to have had an unwanted pregnancy or an Sexually Transmitted Infection [15].

A literature review focussing on the sexual experiences of women with intellectual disabilities found that sex was often a very negative experience. Both women who were and were not having sex, viewed sex as having inevitable negative consequences without any pleasure. It was concluded that their vulnerability and lack of information about sex and consensual relationships had often left women vulnerable to abuse [16].

There is a gap in the research about levels of sexual activity and sexual health amongst young people with intellectual disabilities, and gaps in the knowledge about what socioeconomic factors are associated with sexual activity and sexual health for this group.

Research suggests that socio-demographic variables influence levels of sexual activity and health among adolescents. A large scale study of sexual activity and readiness among adolescents from the general population found a link between sexual activity and socio-demographic factors such as social class, and lower level of maternal education [17]. Another population based study in Ireland found that adolescents were more likely to have had sex if they were older, experimented with alcohol or drugs, were from poorer backgrounds and had lots of friends [18].

This paper presents a secondary analysis of a nationally representative longitudinal survey of young people focussing on sexual activity and sexual health amongst young people with mild/moderate intellectual disabilities. It includes data on social networks and friendships which the previous literature suggests plays a part in influencing sexual knowledge. The research questions were: Are there differences in sexual activity and sexual health between participants with and without intellectual disability? What is the association between intellectual disability and exposure to socio-demographic variables predictive of sexual activity/health outcomes? Which socio-demographic variables are associated with key sexual activity/health outcomes?

## Method

This paper is based on a secondary analysis of data collected in Waves 1 to 7 of *Next Steps* (formerly the Longitudinal Study of Young People in England). *Next Steps* is an annual panel study that followed a cohort from early adolescence into adulthood. It has collected information about their education and employment, economic circumstances, family life, physical and emotional health and wellbeing, social participation and attitudes. *Next Steps* data has also been linked to the Department for Education’s National Pupil Database (NPD). *Next Steps* is currently managed by the Centre for Longitudinal Studies at University College London and is funded by the Economic and Social Research Council. Prior to 2013 it was managed and funded by the Department for Education and NatCen Social Research, with fieldwork conducted by BMRB Social Research, GfK NOP and Ipsos MORI. From 2013 onwards, Next Steps has been funded by the Economic and Social Research Council.

*Next Steps* data files and documentation were obtained from the UK Data Service. Full details of the method and design of *Next Steps* are available in a series of user guides [19]. Key aspects are summarised below.

### Sampling

Fieldwork commenced in 2004 when the sampled children were aged 13–14 (school year 9). The initial (Wave 1) sample was drawn from a sampling frame based on children attending maintained schools, independent schools, special schools and pupil referral units in England who in February 2004 were in Year 9 (or equivalent) and were born between 1 September 1989 and 31 August 1990. Schools in deprived areas and students from minority ethnic groups were oversampled. At Wave 1, 73% of selected schools participated leading to an issued sample of approximately 21,000 young people. The attained sample at W1 was 15,770 children (75% response rate). This cohort was followed-up every year until 2010 (age 19–20).

### Identification of participants with mild/moderate intellectual disability

Data linkage with the 2004 and 2006 NPD was undertaken to identify participants with Special Educational Needs (SEN). Linkage was successful for 15,240 young people present at Wave 1 (97% of the *Next Steps* sample). Linkage included data on stage of assessment and primary/secondary category of Special Educational Needs (SEN).

Following the example of previous studies [20, 21], we used the SEN category of Moderate Learning Difficulty (MLD), if the child was at the School Action Plus stage of assessment of SEN or had a formal Statement of SEN, as an indicator of mild/moderate intellectual disability. School Action Plus and Statements require the involvement of professionals external to the school in the categorisation of SEN. Current guidance defines MLD in relation to pupils having ‘attainments significantly below expected levels in most areas of the curriculum despite appropriate interventions [and having] ... much greater difficulty than their peers in acquiring basic literacy and numeracy skills and in understanding concepts’ [22].

Of the children sampled, 527 (3.5% of the unweighted linked sample) were identified as having mild/moderate intellectual disabilities in either 2004 or 2006. Consistent with the data from existing epidemiological research, the prevalence of mild/moderate intellectual disability was significantly higher among boys than girls (4.3% vs 2.5%; Prevalence Ratio = 1.75(1.46–2.09)) and among children who were eligible for free school meals, an indicator of household poverty, (8.0% vs 1.9%; Prevalence Ratio = 4.10(3.14–5.35)).[1, 23, 24]

### Procedure

Data in the first four waves was collected by face to face interviews using computer assisted personal interviewing with the young person themselves and their parents. Waves 5–7 used a mixed mode approach in which information, which was only collected from the young person, was collected by their choice of method (online, telephone or face to face).

### Measures

#### Sexual activity & sexual health

##### Sexual activity

At Waves 6 and 7 participants were asked; *Have you ever had sexual intercourse with someone?* If their response was yes, they were then asked; *How old were you when you first had sexual intercourse?* We used Wave 7 data to create two binary variables: (1) has had sexual intercourse; (2) sexual intercourse below age 16. If W7 data were missing, responses were used from Wave 6.

##### Unsafe sex

At Waves 6 and 7 participants were asked; *Have you ever had sex without using precautions or contraception? Please do not include any times when you might have been trying for a baby*. If their response was yes, they were then asked; *How often would you say you have sex without using precautions or contraception? Please do not include any times when you might be trying for a baby* (rarely, less than half the time, around half the time, most times, always). We used Wave 7 data to create two binary variables: (1) has had unsafe sex; (2) has unsafe sex around 50% of the time or more. If W7 data were missing, responses were used from Wave 6.

##### Sexually transmitted infections (STI)

At Waves 6 and 7 participants were asked; *Have you ever contracted a sexually transmitted infection (such as Chlamydia, gonorrhoea or genital warts)?* We used Wave 7 data to create a binary variable; has had STI. If W7 data were missing, responses were used from Wave 6.

##### Pregnancy and pregnancy outcomes

At Waves 6 and 7 participants were asked; *Have you ever been pregnant?* We used Wave 7 data to create a binary variable; has been pregnant. If W7 data were missing, responses were used from Wave 6. At Wave 6 only respondents were also asked whether they had the baby and, if not, why (response options: a miscarriage, an abortion, something else). We used Wave 6 data to create two binary variables; had miscarriage, had abortion.

##### Parenting

At Waves 5–7 participants were asked; *Do you have any children of your own?* If their response was yes, they were then asked; *Does this child / Do any of these children currently live in the household with you?*

#### Socio-demographic variables

##### Family socio-economic position

Linkage to the 2004 (Wave 1) and 2006 (Wave 3) NPD included linkage to data on eligibility for free school meals (FSM). Eligibility for FSMs is determined by data linkage to government records of receipt of at least one of a defined list of means-tested welfare benefits by the child’s parent(s). It should be noted that this indicator is of eligibility for, not uptake of, free school meals. We created a binary variable of FSM eligibility scored 1 if the child was eligible at Wave 1, Wave 3 or both Waves of *Next Steps* and scored 0 if the child was not eligible at both Waves. FSM eligibility is a commonly used proxy indicator of low household socio-economic position [25].

We extracted data from *Next Steps* on the employment status of parental figures living in the household at Waves 1–4 inclusive. We created a binary variable of living in a workless household scored 1 if no resident parental figure was in employment or full time education at any of the four Waves and scored 0 if at least one resident parental figure was in employment or full time education in each of the four Waves.

##### Young adult socio-economic position

We extracted data from *Next Steps* on the self-reported employment, education and training status of the young person at Waves 5–7. We created a binary variable of not in employment, education or training (NEET) scored 1 if the young person was not in employment, education or training at any of the three Waves and scored 0 if they were in employment, education or training in each of the three Waves.

##### Household composition

We extracted data from *Next Steps* on household composition at Waves 1–4 inclusive. We created a binary variable of single parent household scored 1 if only one parental figure was resident at any of the four Waves and scored 0 if two parental figures were resident in each of the four Waves.

##### Area deprivation

Linkage to the 2004 (Wave 1) and 2006 (Wave 3) NPD also included linkage to data derived from the postal code of the child’s residence to the Income Deprivation Affecting Children Index (IDACI) [26]. IDACI scores are the percentage of children in each Lower Level Super Output Area (LSOA) that live in families that are considered income deprived. Income deprivation is defined by receipt of means-tested welfare benefits. LSOAs are neighbourhoods with an average population of 1500 (range 1000–3000). IDACI scores were transformed into sample quintiles. We created a binary variable of High Neighbourhood Deprivation scored 1 if the child was living in the lowest IDACI quintile at Wave 1, Wave 3 or both Waves of *Next Steps* and scored 0 if the child was not living in the lowest IDACI quintile at both Waves.

##### Peer victimisation

We extracted data from *Next Steps* on child self-reported experience of peer victimisation (bullying) at Waves 1–3. At each of these waves children were asked about exposure to five types of peer victimisation experienced in the last 12 months:

1. *Have you ever been upset by being called hurtful names by other students, including getting text messages or emails from them?*
2. *Have you ever been excluded from a group of friends or from joining in activities?*
3. *Have other students at your school ever made you give them money or personal possessions?*
4. *Have other students ever THREATENED to hit you, kick you or use any other form of violence against you?*
5. *Have other students ever ACTUALLY hit you, kicked you or used any other form of violence against you?*

If the young participant selected a ‘yes’ option they were then asked about the frequency of exposure (response options: every day, a few times a week, once or twice a week, once every 2 weeks, once a month, less often than this, it varies). Preliminary analysis of responses indicated a strong association between threat of and actual violence, but weak associations between other forms of peer victimisation. As a result we combined self-report of threat of or actual violence at each of the three Waves. For each of the four types of peer victimisation (name calling, social exclusion, theft, violence) we created two binary variables: (1) whether this had happened at all in any 12 month period in Waves 1–3 (contrasted with it having never happened in any of the three Waves); and (2) whether this had happened at all in any 12 month period with at least a weekly frequency in Waves 1–3 (contrasted with it having never happened with this frequency in any of the three Waves).

##### Friendships

We extracted information on friendships from Waves 2, 6 and 7 of *Next Steps*. At Wave 2 participants were asked: *When you have free time, do you mainly: (1) Go out somewhere with friends; (2) Go round to a friend’s house (or friends come round to yours); (3) Spend time with brother(s)/sister(s); (4) Spend time with other members of your family or; (5) Spend time by yourself?* We created a binary variable, W2 spends time with friends, scored 1 if they selected option 1 or 2, scored 0 if they selected options 3–5.

At Waves 6 and 7 participants were asked: *How many close friends do you have – that is friends you could talk to if you were in some sort of trouble?* We created a binary variable, W6/7 few friends, scored 1 if they reported at either Wave they had no or only 1 close friend and scored 0 if they reported at any Wave they had two or more close friends.

### Rate and predictors of sample retention

Retention rates over time are presented in Table 1 for participants with/without intellectual disability.

**Table 1:** Retention rates for participants with/without intellectual disability

Wave	With intellectual disabilities				Without intellectual disabilities		
	N	Unweighted prevalence	% retention from W1	% retention from previous wave	N	% retention from W1	% retention from previous wave
W1	527	3.5%			14,687		
W2	415	3.2%	79%	79%	12,654	86%	86%
W3	354	2.9%	67%	85%	11,649	79%	92%
W4	314	2.8%	60%	89%	10,721	73%	92%
W5	256	2.6%	49%	82%	9551	65%	89%
W6	241	2.6%	46%	94%	8944	61%	94%
W7	206	2.5%	39%	85%	7941	54%	89%

Socio-demographic factors associated with sample attrition between Waves 1 and 7 were examined separately for participants with and without intellectual disability. Predictors of attrition were broadly similar for participants with and without intellectual disability, with male gender, membership of a minority ethnic group, household poverty (defined by free school meal eligibility) and higher neighbourhood deprivation all being associated with higher rates of attrition [27, 28]. For the variables ‘boys’ and ‘not White British’ the point estimate for attrition in the non-intellectual disability group (boys 1.10 (1.07–1.15); not White British 1.17 (1.13–1.22)) lay within the 95% CI of the intellectual disability group (boys 1.28 (1.09–1.51)); not White British 1.12 (0.97–1.29)). For the variables ‘eligibility of free school meals and ‘high neighbourhood deprivation’ the point estimate for non-participation in the non-intellectual disability group (eligibility of free school meals 1.37 (1.32–1.43); high neighbourhood deprivation 1.38 (1.30–1.40)) was greater than the upper 95% CI of the intellectual disability (eligibility of free school meals 1.15 (1.00–1.33); high neighbourhood deprivation 1.09 (0.94–1.26)).

### Approach to analysis

In the first stage of analysis we made simple bivariate comparisons (prevalence ratios) between participants with and without intellectual disability with regard to available indicators of sexual activity and sexual health.

In the second stage of analysis we investigated, for key indicators of sexual activity and sexual health, the strength of association between socio-demographic factors and outcomes separately for participants with and without intellectual disability. Missing data among socio-demographic variables was imputed using multiple imputation routines in SPSS 22 to create five parallel imputed data sets. Poisson regression with robust standard errors was used to estimate prevalence ratios uniquely associated with each variable in the model [29, 30]. The subsequent analysis used the following approach: (1) five blocks of variables were created (SEP, neighbourhood, family type, peer victimisation, friendships) and entered sequentially; (2) variables within blocks were entered in order of bivariate strength of association with the outcome of interest; (3) variables were only retained in the model if *at the point of entry* they were significantly related to the outcome of interest or had a prevalence ratio of 1.50 or greater.

In the final stage of analysis we estimated the strength of association between intellectual disability and sexual activity/health outcomes while controlling for between group differences in exposure to socio-demographic variables that have been established as important social determinants of poorer health. We used Propensity Score Matching routines in SPSS 22 to match each participant with intellectual disability with a participant without intellectual disability with a similar propensity score for intellectual disability based on exposure to the socio-demographic variables [31–33]. We used the lowest tolerance for matching (0.05) that allowed complete matching for all participants with intellectual disability.

## Results

### Are there differences in sexual activity and sexual health between participants with and without intellectual disability?

Table 2 shows that overall, people with intellectual disabilities were less likely to have had sexual intercourse by age 19/20 than their peers. However, if they were sexually active then: (1) girls with intellectual disability were significantly less likely than other girls to have had their first experience of sexual intercourse below the age of 16; (2) boys and girls with intellectual disability were significantly more likely to commonly have unsafe sex; (3) girls with intellectual disability were more likely to have been pregnant; and (4) were more likely to be mothers.

**Table 2:** Sexual activity and sexual health among participants with and without intellectual disability

	Sex	Participants with intellectual disabilities		Other participants		Unadjusted Prevalence Ratio
		Total n	%	Total n	%	
Sexually Active						
Ever had sexual intercourse	Men	186	75%	4150	89%	0.84*** (0.77–0.92)
	Women	115	72%	4287	88%	0.82*** (0.73–0.92)
First had sexual intercourse below age 16	Men	129	41%	3488	36%	1.12 (0.91–1.26)
	Women	76	15%	3555	33%	0.45** (0.26–0.77)
Unsafe sex						
Ever had unsafe sex	Men	139	58%	3673	56%	1.03 (0.89–1.19)
	Women	81	59%	3746	51%	1.16 (0.97–1.40)
Has unsafe sex on 50% time or more instances	Men	137	26%	3634	18%	1.39* (1.03–1.86)
	Women	78	22%	3710	13%	1.71* (1.12–2.63)
STI						
Ever	Men	110	4%	3335	4%	1.05 (0.39–2.79)
	Women	69	4%	3463	7%	0.61 (0.20–1.84)
Pregnancies & Children						
Ever	Women	76	50%	3709	23%	2.17*** (1.72–2.74)
Abortion	Women	39	8%	856	23%	0.33* (0.11–0.99)
Miscarriage	Women	39	5%	856	13%	0.40 (0.10–1.56)
Has child at 17/18	Men	210	0%	4565	1%	0.20 (0.01–3.20)
	Women	119	12%	4716	4%	2.95*** (1.77–4.92)
Has child at 18/19	Men	197	2%	4270	3%	0.61 (0.20–1.90)
	Women	122	18%	4316	7%	2.54*** (1.72–3.77)
Has child at 19/20	Men	167	7%	3777	4%	1.55 (0.86–2.79)
	Women	105	25%	3871	10%	2.58*** (1.83–3.66)
Child lives elsewhere at 19/20	Men	11	73%	210	58%	1.25 (0.86–1.83)
	Women	20	0%	20	2%	0.25 (0.00–126.81)

* *P*<0.05 **P<0.01 and *** *P*<0.001

### What is the association between intellectual disability and exposure to socio-demographic variables predictive of sexual activity/health outcomes?

Table 3 illustrates that participants with intellectual disabilities were significantly more likely to be exposed to all socio-demographic indicators associated with poorer health outcomes (e.g., low family socio-economic position, living in a more deprived area, exposure to violence) than participants without intellectual disabilities.

**Table 3:** Association between intellectual disability and exposure to socio-demographic variables predictive of sexual activity/health outcomes

	% PWID	% Others	Prevalence Ratio adjusted for sex
Socio-Economic Position			
FSM eligible W1 or w3	45%	17%	2.82^***^ (2.52–3.17)
Workless HH W1–4 (any wave)	48%	19%	2.77^***^ (2.50–3.08)
NEET W5–7 (any wave)^a^	38%	15%	2.40^***^ (2.09–2.75)
Household Composition			
Single parent household W1–4 (any wave)	46%	30%	1.58^***^ (1.42–1.75)
Neighbourhood			
Lowest Q of IDACI W1 or W3	30%	16%	2.02^***^ (1.73–2.36)
Friendships			
Spare time mainly spent with friends (W2)	56%	75%	0.70^***^ (0.64–0.77)
No or only 1 close friend (W6 or W7)^a^	20%	8%	2.61^***^ (2.09–3.27)
Peer Victimisation (W1–3 any wave)			
Threatened with violence/attacked	51%	40%	1.26^***^ (1.15–1.38)
Threatened with violence/attacked at least weekly	33%	21%	1.50^***^ (1.31–1.72)
Robbed	16%	6%	3.00^***^ (2.41–3.74)
Robbed at least weekly	13%	4%	3.46^***^ (2.69–4.46)
Called names etc. ….	56%	41%	1.51^***^ (1.39–1.64)
Called names etc. …. at least weekly	40%	24%	1.86^***^ (1.66–2.09)
Socially excluded	43%	30%	1.58^***^ (1.42–1.76)
Socially excluded at least weekly	33%	19%	1.88^***^ (1.65–2.14)

Notes:

Data weighted using W1 cross-sectional rates unless specified

^a^ Data weighted using W5–7 cross sectional weights

^***^ *p* < 0.001

### Which socio-demographic variables are associated with key sexual activity/health outcomes?

Table 4 shows the results of multivariate analyses (Poisson regression with robust standard error) to identify unique predictors of four sexual activity/health outcomes separately for participants with/without intellectual disability.

**Table 4:** Predictors of key sexual activity/health outcomes for participants with and without intellectual disability

Outcome/Group	Variable	People with ID PR (95% CI)	People without ID PR (95% CI)
Women: Unsafe sex on 50% + of occasions	FSM eligibility	1.32 (0.55–3.17)	0.97 (0.77–1.21)
	Workless household		1.64*** (1.30–2.07)
	NEET		1.85*** (1.52–2.24)
	Single parent HH		1.23* (1.01–1.49)
	Bullied weekly (names)	3.61* (1.30–10.01)	
	Bullied (robbed)		1.34* (1.01–1.77)
	Bullied (threat of or actual violence)		1.45*** (1.22–1.74)
	W2 spare time spent with friends	2.50* (1.01–6.14)	1.33* (1.05–1.68)
	W6/7 0 or 1 close friend	2.78** (1.41–5.47)	
Men: Unsafe sex on 50% + of occasions	FSM eligibility		1.33** (1.12–1.59)
	NEET	2.74** (1.46–5.13)	1.37*** (1.16–1.61)
	High neighbourhood deprivation		1.37** (1.14–1.64)
	Bullied (robbed)	1.82* (1.09–3.05)	
	Bullied weekly (robbed)		1.32 (1.00–1.75)
	Bullied weekly (socially excluded)		1.41*** (1.22–1.66)
	W2 spare time spent with friends		1.44*** (1.18–1.76)
Ever pregnant	FSM eligibility	1.83** (1.22–2.75)	1.41*** (1.19–1.68)
	Workless household		1.24* (1.05–1.47)
	High neighbourhood deprivation		1.29** (1.12–1.49)
	Single parent HH		1.39*** (1.22–1.59)
	Bullied (threat of or actual violence)		1.30*** (1.15–1.48)
	Bullied (socially excluded)		1.15* (1.02–1.31)
	W2 spare time spent with friends	2.11** (1.27–3.49)	1.34*** (1.14–1.58)
	W6/7 0 or 1 close friend		1.82*** (1.58–2.11)
Mother at 17/18	Workless household	2.99*** (2.07–4.33)	3.51*** (2.41–5.09)
	High neighbourhood deprivation	1.29 (0.88–1.87)	
	Single parent HH	2.32*** (1.59–3.40)	2.28*** (1.51–3.38)
	Bullied (robbed)	1.77* (1.14–2.74)	
	Bullied weekly (robbed)		0.90 (0.47–1.75)
	Bullied weekly (socially excluded)		1.54* (1.07–2.22)
	Bullied (threat of or actual violence)		1.81** (1.28–2.57)
	W2 spare time spent with friends	1.85** (1.24–2.78)	1.70* (1.12–2.56)
	W6/7 0 or 1 close friend	2.43*** (1.69–3.49)	2.38*** (1.63–3.48)
Mother at 19/20	FSM eligibility		1.92*** (1.46–2.52)
	Workless household	1.93*** (1.54–2.42)	1.27 (0.97–1.67)
	High neighbourhood deprivation	1.38** (1.10–1.74)	1.30* (1.03–1.64)
	Single parent HH	1.88*** (1.52–2.32)	1.85*** (1.50–2.30)
	Bullied weekly (socially excluded)		1.24* (1.00–1.53)
	Bullied (threat of or actual violence)		1.44** (1.18–1.75)
	W6/7 0 or 1 close friend	2.00*** (1.61–2.49)	2.05*** (1.63–2.57)

Note: Empty cells indicate variable was not retained in the model

* *P*<0.05 **P<0.01 and *** *P*<0.001

In particular the association between many variables (all family SEP variables, family type, early neighbourhood deprivation) and all four outcomes was stronger among people without intellectual disabilities. Young motherhood was associated with indicators of low family socio-economic position, family type, and early neighbourhood deprivation. Both men and women with intellectual disabilities who were bullied were more likely to report unsafe sex on 50% + of occasions. Men with intellectual disabilities who were not in Education, employment or training (NEET) were also more likely to report unsafe sex on 50% + occasions.

### Are between-group differences in sexual activity/health apparent when controlling for between-group differences in exposure to socio-demographic variables predictive of poorer outcomes?

Table 5 shows that adjusting for between group differences in exposure to socio-demographic variables had no impact on the association between intellectual disability and ever having had sexual intercourse and age of first having sexual intercourse. Adjusting markedly increased the risk associated with intellectual disability for three outcomes (adjusted point estimate was greater than upper 95% CI for unadjusted estimate: male unsafe sex on 50% or more occasions, motherhood by age 19/20, men having a child living elsewhere). There were also trends for increased risk associated with intellectual disability to for two additional outcomes (women having unsafe sex on 50% or more occasions, men having a STI).

**Table 5:** Sexual activity and sexual health outcomes for participants with and without intellectual disability adjusted for differential exposure to socio-demographic variables

	Sex	Unadjusted Prevalence Ratio	Prevalence Ratio for propensity score matched groups (tolerance 0.05)
Sexually Active			
Ever had sexual intercourse	Men	0.84*** (0.77–0.92)	0.82** (0.72–0.90)
	Women	0.82*** (0.73–0.92)	0.86 (0.73–1.01)
First had sexual intercourse below age 16	Men	1.12 (0.91–1.26)	0.87 (0.60–1.26)
	Women	0.45** (0.26–0.77)	0.51 (0.25–1.04)
Unsafe sex			
Ever had unsafe sex	Men	1.03 (0.89–1.19)	1.13 (0.86–1.48)
	Women	1.16 (0.97–1.40)	1.32 (0.97–1.81)
Has unsafe sex on 50% time or more instances	Men	1.39* (1.03–1.86)	2.21* (1.10–4.44)
	Women	1.71* (1.12–2.63)	2.15* (1.09–4.28)
STI			
Ever	Men	1.05 (0.39–2.79)	2.21 (0.38–12.90)
	Women	0.61 (0.20–1.84)	0.37 (0.08–1.60)
Pregnancies & Children			
Ever	Women	2.17*** (1.72–2.74)	2.45*** (1.51–3.97)
Abortion	Women	0.33* (0.11–0.99)	0.28 (0.03–2.50)
Miscarriage	Women	0.40 (0.10–1.56)	0.84 (0.13–5.46)
Has child at 17/18	Men	0.20 (0.01–3.20)	n/a 0% in both groups
	Women	2.95*** (1.77–4.92)	2.50 (0.84–7.44)
Has child at 19/20	Men	1.55 (0.86–2.79)	1.11 (0.33–3.74)
	Women	2.58*** (1.83–3.66)	3.70** (1.59–8.63)
Child lives elsewhere	Men	1.25 (0.86–1.83)	5.82* (0.90–37.70)
	Women	0.25 (0.00–126.81)	n/a 0% in both groups

* *P*<0.05 **P<0.01 and *** *P*<0.001

To further explore the results from Propensity Score Matching we undertook sensitivity analysis using different tolerances for matching (0.01, 0.1, 0.2). At two of the three additional tolerances (0.1, 0.2) there was a marked increase in the risk associated with intellectual disability for men having unsafe sex on 50% or more occasions and having a child living elsewhere. At none of the three additional tolerances there was a marked increase in the risk associated with intellectual disability for motherhood at age 19/20.

## Discussion and conclusions

The findings from this nationally representative sample are consistent with previous small scale research that suggested young people with mild/moderate intellectual disabilities were less likely to have had sexual intercourse than the general population, but if they were sexually active they were more likely to have engaged in unsafe sex [15]. This population sample was taken from UK mainstream schools where sex and relationships education are a compulsory part of the curriculum, therefore all of the young people in this cohort would have received a sex education programme.

Young people with mild/moderate intellectual disabilities were more likely to have experienced a range of social and material disadvantages (low family socio-economic position, living in a more deprived area, exposure to violence) than those without intellectual disabilities. This echoes previous research around socioeconomic position [11]. Again in keeping with previous literature, peer victimisation was also significantly higher for young people with mild/moderate intellectual disabilities [12]. They experienced more episodes of violence (both threats and being attacked), theft, weekly theft, being called names and being socially excluded. They also reported more often than those in the general population that they had only one or no friends. In previous studies a lack of friendship and opportunity to discuss sex topics with friends is linked to a lack of consolidation and understanding of issues around sexual health [10].

The implications for this study are that Education and health services, and families, need to operate on the assumption that most young people with mild/moderate intellectual disabilities will have sex, therefore education and interventions concerning sexual activity and sexual health need to be accessible to and effective for adolescents with intellectual disabilities. This research suggests that young people with intellectual disabilities in mainstream schools may not be responding to sex education in the same way as their peers. Young people from the general population were more likely to use contraception, compared with those with intellectual disabilities. This may be due to the lack of social networks, reduced opportunity to talk to friends and consolidate knowledge [4]. Social networks are moving increasingly online which may exclude people with intellectual disabilities whose lack of literacy skills may make it difficult to communicate online [34, 35]. Furthermore, if there is a reluctance from families to discuss sex and contraception with young people then they again are less likely to consolidate their existing knowledge [5]. Educators and health professionals therefore should take these findings on board when looking at policies around young people with intellectual disabilities and sex education.

The rates of multiple instances of unsafe sex (50% or more instances) were lower for those from the general population than for adolescents with intellectual disabilities. This illustrates that young people with intellectual disabilities were more likely to have unsafe sex regularly, than young people from the general population. Sexual health clinics need to be equipped to deal with people with mild/moderate intellectual disabilities. Repeated episodes of unsafe sex put young people at a greater risk of pregnancy and STIs, and in this study pregnancy rates were higher for the young people with intellectual disabilities. Again this echoes the findings of McCabe and Cummins [15] who found that people with mild ID were more likely to have had a STI or unwanted pregnancy despite being less likely to have had sexual experiences or intercourse than other adolescents.

### Strengths & limitations

The strengths of this study are that it is large in scale compared to other research in this area, the research uses a nationally representative sampling frame, and it is longitudinal. The use of cross-sectional weights takes account of the original sample design and biases in initial recruitment and retention due to such factors as gender and multiple indicators of socio-economic position to ensure that the weighted analyses are representative of the target population.

Mild/moderate intellectual disability was ascertained from educational administrative status (SEN of MLD). While this categorization shows expected associations with gender and socio-economic disadvantage and provides similar prevalence rates to mild/moderate intellectual disability [24], the degree of correspondence between the two constructs has not been formally validated.

The limitations of this study are due to the nature of undertaking secondary analysis, such as being reliant on the questions asked in the study. There were high rates of attrition for the Next Steps study, particularly amongst young people with intellectual disabilities. This unfortunately meant that, as there was no over-sampling of children with intellectual disabilities, there are small numbers for some analyses.

Using free school meals as a measure of disadvantage can be controversial due to the underestimation of the ‘working poor’, and those who are ineligible but live in poverty [25]. This was only one of the measures of disadvantage used in the study, however, clearly there will be some young people living in difficult socio-economic circumstances who are not included in the FSM data.

The participants in the study were interviewed face to face for the first wave and then followed up at different waves using a mixed mode approach in which information was collected by their choice of method (online, telephone or face to face). Young people may have been reluctant to be truthful due to embarrassment or stigma surrounding some of the sexual topics, for example whether they have ever had a STIs [36, 37]. There were low numbers of STIs reported in the study, especially considering the high rate of unsafe sex. It is worth remembering that many STIs remain symptomless and unless tested, participants may be unaware they have contracted an STI [38, 39].

In conclusion the study addresses the gap in the literature regarding sexual activity and sexual health of young people with mild to moderate intellectual disabilities. It highlights that young people with mild/moderate intellectual disabilities are likely to be sexually active by age 19/20 and therefore education and health services need to operate on this assumption. Within mainstream schools, young people with intellectual disabilities are receiving sex education but this is not preventing their engagement in unsafe sex.

## Abbreviations

AOR: Adjusted odds ratio
IQ: Intelligence quotient
